# Supplementary material for: Antiparallel protocadherin homodimers use distinct affinity- and specificity-mediating regions in cadherin repeats 1-4
Source: eLife. 2016 Jul 29;5:e18449. doi: 10.7554/eLife.18449 (PMC5001838; doi:10.7554/eLife.18449)
Supplement: Figure 1—source data 1. — DOI: http://dx.doi.org/10.7554/eLife.18449.004 [file elife-18449-fig1-data1.docx]

| PDB ID | 5K8R |
| --- | --- |
| SBGridDB | 325 |
| Data Collection |  |
| Beam source | APS 24-ID-E |
| Wavelength (Å) | 0.97918 |
| Space group | C222_1_ |
| Unit cell (a, b, c; Å) | 128.9, 161.2, 53.7 |
| Resolution (Å) | 47.4 ­ 2.5 (2.6 ­ 2.5) |
| Total reflections | 135392 (13162) |
| Unique reflections | 19844 (821) |
| Multiplicity | 6.8 (6.7) |
| Completeness (%) | 92 (42) |
| Mean I/σ(I) | 10.1 (1.0) |
| Resolution shell at I/σ(I) = 2 (Å) | 2.72 - 2.64 |
| Wilson B-factor | 37.27 |
| R_merge_ | 0.133 (1.96) |
| R_meas_ | 0.145 (2.13) |
| CC_1/2_ | 0.998 (0.414) |
| CC* | 1 (0.765) |
| Refinement |  |
| Refinement resolution range | 47.4 ­ 2.5 (2.6 ­ 2.5) |
| Reflections used in refinement | 18203 (821) |
| Reflections used for R-free | 1814 (85) |
| R_work_ | 0.205 (0.271) |
| R_free_ | 0.258 (0.336) |
| CC_work_ | 0.908 (0.743) |
| CC_free_ | 0.850 (0.534) |
| Number of non-hydrogen atoms | 3370 |
| Macromolecules | 3219 |
| Ligands (Ca^2+^, Cl^-^, Na^+^, HEPES, ethylene glycol) | 31 (9, 2, 1, 15, 4) |
| Waters | 120 |
| Protein residues | 414 |
| RMS |  |
| Bonds (Å) | 0.006 |
| Angles (°) | 0.62 |
| Clashscore | 9.18 |
| Average B-factor | 47.2 |
| Macromolecules | 47.2 |
| Ligands | 66.7 |
| Solvent | 42.8 |
| Ramachandran plot regions |  |
| Favored (%) | 97 |
| Allowed (%) | 2.7 |
| Outliers (%) | 0 |
| Rotamer outliers (%) | 0.55 |
